# Analysis of Tensile Strength and Failure Mechanism Based on Parallel Homogenization Model for Recycled Concrete

**DOI:** 10.3390/ma15010145

**Published:** 2021-12-25

**Authors:** Yijiang Peng, Semaoui Zakaria, Yucheng Sun, Ying Chen, Lijuan Zhang

**Affiliations:** Key Laboratory of Urban Security and Disaster Engineering, Ministry of Education, Beijing University of Technology, Beijing 100124, China; pengyijiang@bjut.edu.cn (Y.P.); Semaouizakaria@emails.bjut.edu.cn (S.Z.); zhanglijuan@bjut.edu.cn (L.Z.)

**Keywords:** mesoscopic damage, recycled concrete, parallel homogenization model, base force element method

## Abstract

In this paper, a parallel homogenization model for recycled concrete was proposed. A new type of finite element method, the base force element method, based on the complementary energy principle and the parallel homogenization model, is used to conduct meso-level damage research on recycled concrete. The stress–strain softening curve and failure mechanism of the recycled concrete under uniaxial tensile load are analyzed using the nonlinear damage analysis program of the base force element method based on the parallel homogenization model. The tensile strength and destructive mechanisms of recycled concrete materials are studied using this parallel homogenization model. The calculation results are compared with the results of the experiments and meso-level random aggregate model analysis methods. The research results show that this parallel homogenization analysis method can be used to analyze the nonlinear damage analysis of recycled concrete materials. The tensile strength, stress–strain softening curve, and crack propagation process of recycled concrete materials can be obtained using the present method.

## 1. Introduction

Recycled aggregates are aggregates made from waste concrete through a series of processing methods. Recycled aggregate concrete is concrete made by replacing part, or all, of the natural aggregate with recycled aggregate. It has been widely valued as a green and environmentally friendly building material [1].

Many scholars have carried out a lot of experimental research on recycled concrete, and some research results have been obtained [1,2,3,4,5]. However, the test cycle is long, the cost is high, and it is difficult to measure the internal stress, strain, and failure mechanisms of the material. Therefore, it is very useful to carry out numerical simulation research on recycled concrete. In reference [6], a plastic-damage constitutive models are employed in numerical studies on recycled concrete under uniaxial compression and uniaxial tension loadings to pre-dict the overall mechanical behavior, particularly the stress–strain relationship. In reference [7], a statistical analysis on its composition has been performed considering the randomness in properties of old adhered mortar around recycled aggregate. Peng et al. [8,9,10,11] proposed the base force element method and used this new type of finite element method to carry out a numerical simulation analysis on the recycled concrete, and conducted uniaxial tensile and compression tests under static and dynamic loads to study its mechanics and performance. In 2016, Rajendra [12] established a virtual crack model and a double-K fracture model, and determined the fracture parameters of recycled concrete with different coarse aggregate contents. In reference [13], a stochastic elastic FEM analyses model was established based on the Nano-indentation technique for recycled concrete at three different scales to obtain the effective elastic moduli and Poisson's ratios, also the correlations of recycled concrete were studied. Anuruddha [14] investigated the influence of the old mortar content on the elastic module and the strength of recycled concrete, and found that the pressure strength of recycled concrete decreased with the increase in mortar content. Job et al. [15] used numerical simulation and regression analysis methods to study various mechanical properties of recycled concrete, and obtained the influence of different replacement rates of recycled aggregate on the strength of recycled concrete. In 2019, Tan [16] carried out a two-dimensional numerical simulation of recycled concrete based on the discrete element method, and mainly studied the influence of the weak link between the old and new interfaces on the damage and failure process of recycled concrete. Guo [17] has developed a creep coupling model for the heterogeneity of recycled concrete, to investigate the influence of recycled aggregate on the creeping of recycled concrete. Kazemian et al. [18] conducted experimental research on the compressive strength, flexural strength, and fracture energy of recycled concrete, and compared the mechanical properties of treated and untreated recycled concrete. There are also some scholars who have carried out research works in this field or other types of concrete [19,20,21,22,23,24,25,26].

In the paper, a homogenization analysis method will be used to establish a parallel homogenization model for recycled concrete materials. A new type of finite element method, the base force element method based on the complementary energy principle, is used to conduct meso-level damage research on recycled concrete. The stress–strain softening curve and failure mechanisms of recycled concrete under uniaxial tensile load are analyzed using the nonlinear damage analysis program of the base force element method.

## 2. Materials and Methods

### 2.1. Random Aggregate Model

**Figure 1:**
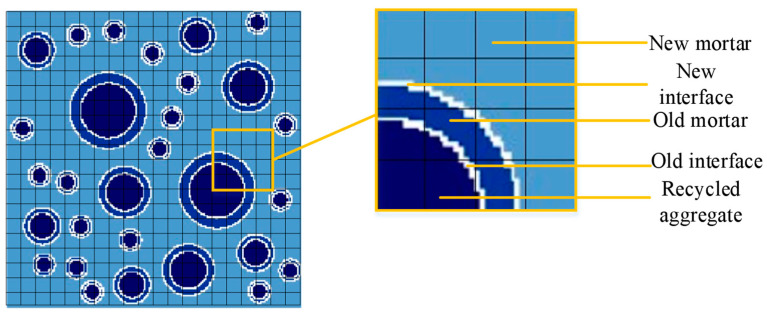
Each phase of random aggregate.

The main difference between natural concrete and recycled concrete is that the outer layer of recycled concrete aggregate is attached with a layer of old mortar. The random aggregate model represents the recycled concrete in the form of each phase medium, so as to facilitate the subsequent mesh division and calculation at the meso level. The circular aggregate model was adopted to simplify the recycled aggregate into two concentric circles, as shown in Figure 1. The macro-mechanical properties of the whole structure are obtained through the analysis and calculation of the random aggregate model. It is the link between the macro-structure and the macro-mechanical properties of recycled concrete.

In Figure 1, the recycled concrete is treated as a 5-phase medium, including the aggregate, the old mortar, the new mortar, the old interface and the new interface. The aggregate center position is automatically generated by a computer program according to the Monte Carlo method. The aggregate size and particle number are calculated according to the grading of recycled concrete. The placement of aggregate should ensure that each aggregate cannot overlap.

The calculation and analysis are carried out on the basis of the random aggregate model of recycled concrete. First, the two-dimensional random aggregate is generated. After that, the mesh is divided and mapped to the model, the position of the element node is judged, and the attributes of each element are assigned. Aggregates are divided into coarse aggregates and fine aggregates. Fine aggregates refer to aggregate particles with a particle size of less than 5 mm. The influence of the fine aggregate is ignored, and the fine aggregate and mortar are regarded as a whole in the research of this article. The data of the particle size range of the coarse aggregate are obtained based on the experimental data.

### 2.2. Parallel Homogenization Model

#### 2.2.1. Parallel Homogenization Model

**Figure 2:**
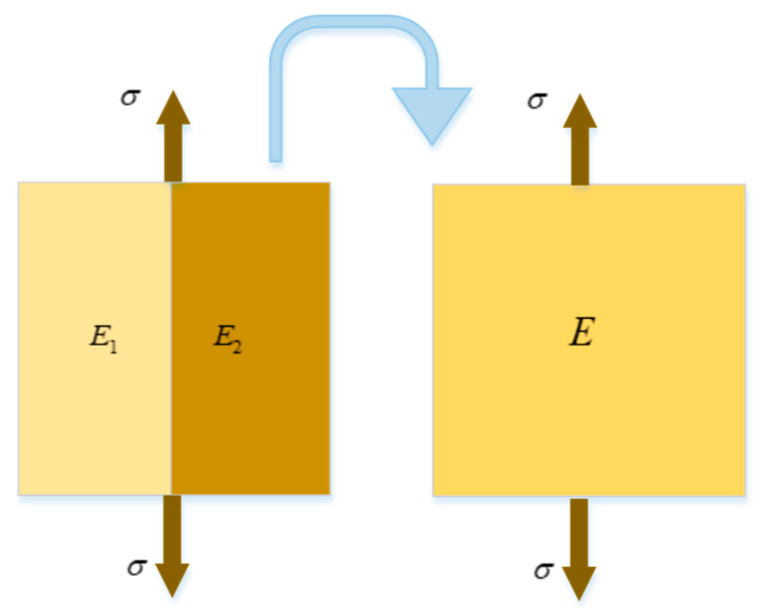
Voigt parallel model.

A parallel method was used to homogenize the heterogeneous elements of recycled concrete, based on the basic idea of equivalent model of meso-damage element, and the equivalent model of meso-damage was established. The validation and numerical calculation of homogenized equivalent model are based on random aggregate model. A simple material strength equivalent method was adopted using the Kelvin–Voigt parallel model, ignoring transverse deformation, as shown in Figure 2. σ is the stress and E is the modulus of elasticity.

The equilibrium equation of element stress is as follows:

(1) $$\sigma A=\sigma_{1}A_{1}+\sigma_{2}A_{2}$$

where *A* is the area of an equivalent element, *A*_1_ and *A*_2_ are the areas of two different media elements, respectively.

The strain compatibility equation is as follows:

(2) $$\varepsilon =\varepsilon_{1}=\varepsilon_{2}$$

The mean stress–mean strain relationship is as follows:

(3) σ=Eε

It can be obtained from the following formulas:

(4) $$E\varepsilon A=E_{1}\varepsilon_{1}A_{1}+E_{2}\varepsilon_{2}A_{2}$$

(5) $$EA=E_{1}A_{1}+E_{2}A_{2}$$

For the composite recycled concrete model, it can used the volume fraction to calculate $EV=E_{1}V_{1}+E_{2}V_{2}$.

Let $c_{1}=\frac{V_{1}}{V}$, $c_{1}=\frac{V_{1}}{V}$ ($c_{1}+c_{2}=1$), then we can obtain the following formula:

(6) $$E=E_{1}c_{1}+E_{2}c_{2}$$

where *c*_1_ and *c*_2_ are the percentage of the two different media elements to the total volume of the equivalent elements, respectively.

The equivalent Poisson’s ratio is as follows:

(7) $$\upsilon =\upsilon_{1}c_{1}+\upsilon_{2}c_{2}$$

#### 2.2.2. Finite Element Meshing and Homogenization

The finite element method is the process of dividing a continuous object into simple and regular elements. It is an effective method for calculating and analyzing objects with heterogeneous materials. Scholars have proposed many meshing methods, as the finite element method is widely used in the scientific community. The most common elements are as follows: one-dimensional rod element, two-dimensional triangle element, quadrilateral element and three-dimensional tetrahedron element, pentahedron element and hexahedron element. The finer and denser the mesh, the more accurate the calculation result for the traditional finite element method. However, at the same time, the more computational elements, the lower the computational efficiency. 

**Figure 3:**
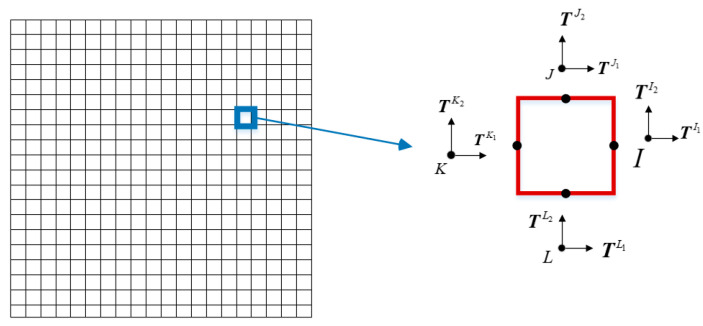
Mesh subsection diagram.

In this paper, the meshing method of the two-dimensional quadrilateral element is selected based on the base force element method of complementary energy principle. The mesh is divided according to the size of the specimen and the element size. The midpoint of each side of the quadrilateral element is used as the calculation point, as shown in Figure 3. Then the mesh is mapped to the random aggregate model.

**Figure 4:**
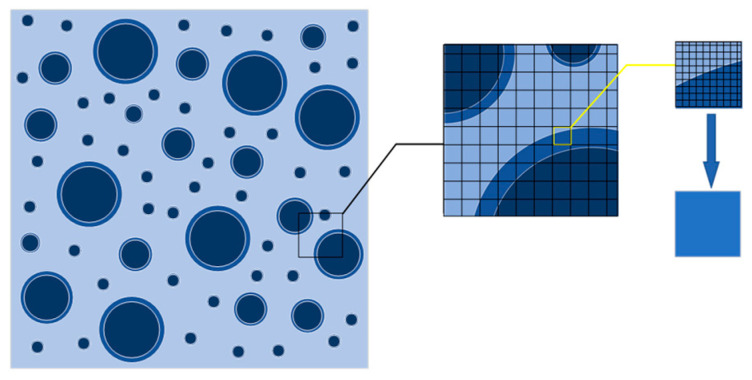
Mesoscopic model of regenerated concrete meshes.

In Figure 4, the fine mesh of the random aggregate meso-model is divided first, and then the coarse mesh is formed by homogenization. The division method and judgment rule of coarse mesh are the same as those of fine mesh, and the judgment rule is determined according to the location of element nodes. The attribute of the element is judged as aggregate (or old mortar, or new mortar), when more than or equal to three of the four nodes of an element are projected on aggregate (or old mortar, or new mortar) medium. The element is defined as old interface element, when some element nodes fall in aggregate medium and some fall in old mortar. Similarly, the element is defined as the new interface when some nodes of an element fall on the old mortar medium and some nodes fall on the new mortar medium. After determining the attributes of fine elements, the number of small elements of each attribute was counted, the area was calculated according to the element size, and the proportion of each component in the coarse mesh element was calculated. The coarse mesh is equivalent to uniform single-attribute element by homogenization method.

The mesh division and attribute assignment of the elements were programmed to calculate, and the mesh node numbers and coordinates of all the specimens were obtained using Fortran language program. The attribute distribution of an element could be obtained from the element attribute file by the calculating program. The composition of the heterogeneous element containing heterogeneous medium could be obtained. The data are provided for the subsequent homogenization calculation.

The divided elements are mapped to the random aggregate model and then each large element is subdivided to obtain small elements. The proportions of the attributes of the small elements, the number of components, and equivalent parameter calculations are determined. The equivalent parameters are assigned to large elements.

**Figure 5:**
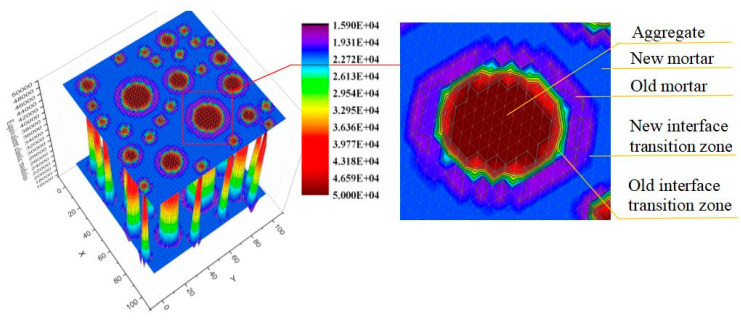
Equivalent elastic modulus distribution.

The distribution of equivalent elastic modulus in the model is obtained. The equivalent elastic modulus distribution of the parallel equivalent method of the test specimen is obtained, as shown in Figure 5.

In the red area of Figure 5, all elements are aggregate. Therefore, the computer’s automatic judgment belongs to the same medium. Computers do not use equivalent processing, that is, the elastic modulus is the elastic modulus of aggregate.

In the blue area in Figure 5, all elements are new mortar. Therefore, after the automatic judgment of the computer, the area belongs to the same medium. Computers do not perform the equivalent, that is, the elastic modulus is the elastic modulus of new mortar.

If all small elements inside the large element (equivalent elements) are old mortar, the area belongs to the same medium. There is no equivalent processing, that is, the elastic modulus is the elastic modulus of old mortar. This is shown in purple in Figure 5.

Equivalent treatment is required if small elements within large elements have aggregate elements and interface elements, or if large elements contain aggregate elements, mortar elements and interface elements. The old interface transition zone and new interface transition zone belong to equivalent elements, as shown in Figure 5. The elastic modulus varies in this region. There are different colors.

#### 2.2.3. Damage Model

The stress–strain relationship of material is a very important and complex problem in the case of material damage. The constitutive nature of material is an old and still open question, starting with the papers of Hudson et al., Bažant and Chang [27,28,29]. Even more recently, Ferretti [30] has shown that the meso-scale curves (mean stress/mean strain and damage curves) are not constitutive, while it is possible to identify constitutive laws at the micro scale.

In this paper, the multi-line stress–strain relationship is adopted for calculation because the stress tends to be highly nonlinear when approaching the peak value under uniaxial tensile load, due to the non-uniformity of the recycled concrete. The material is damaged due to stretching. By introducing the scalar damage variable D, the relationship between the effective strain of the damaged material and Cauchy stress is as follows:

(8) $$\sigma =E_{0}(1-D)\varepsilon$$

The elastic modulus after damage can be expressed by the initial elastic modulus, if the effect of damage on Poisson’s ratio is neglected, as follows:

(9) $$E=E_{0}(1-D)$$

where E represents the elastic modulus after damage, and $E_{0}$ represents the initial elastic modulus. Therefore, the damage elastic modulus of the five-phase medium in recycled concrete can be expressed as (aggregate (ag), old mortar (om), new mortar (m), old interface (oitz), new interface (itz)).

(10) $$\left\{ \begin{array}{l} E^{ag}=E_{0}{}^{ag}(1-D^{ag})\\ E^{om}=E_{0}{}^{om}(1-D^{om})\\ E^{m}=E_{0}{}^{m}(1-D^{m})\\ E^{oitz}=E_{0}{}^{oitz}(1-D^{oitz})\\ E^{\mathrm{itz}}=E_{0}{}^{itz}(1-D^{itz}) \end{array}\right.$$

(11) $$D_{t}=\{\begin{array}{ccc} 0 & & \varepsilon_{\max}\leq \varepsilon_{t0}\\ 1-\frac{\varepsilon_{t0}}{\varepsilon_{\max}}+\frac{\varepsilon_{\max}-\varepsilon_{t0}}{\eta_{t}\varepsilon_{t0}-\varepsilon_{t0}}\frac{\varepsilon_{t0}}{\varepsilon_{\max}}\left(1-\mu \right) & & \varepsilon_{t0}<\varepsilon_{\max}\leq \eta_{t}\varepsilon_{t0}\\ 1-\frac{\mu }{\xi_{t}-\eta_{t}}\frac{\varepsilon_{\max}-\eta_{t}\varepsilon_{t0}}{\varepsilon_{\max}}+\frac{\mu \varepsilon_{t0}}{\varepsilon_{\max}} & & \eta_{t}\varepsilon_{t0}<\varepsilon_{\max}\leq \xi_{t}\varepsilon_{t0}\\ 1 & & \varepsilon_{\max}>\xi_{t}\varepsilon_{t0} \end{array}$$

(12) $$D_{c}=\{\begin{array}{ccc} 1-\frac{\delta }{\omega } & & \varepsilon_{\max}\leq \lambda \varepsilon_{c0}\\ 1-\frac{1-\delta }{1-\lambda }\frac{\varepsilon_{\max}-\lambda \varepsilon_{co}}{\varepsilon_{\max}}-\delta \frac{\varepsilon_{co}}{\varepsilon_{\max}} & & \lambda \varepsilon_{c0}<\varepsilon_{\max}\leq \varepsilon_{c0}\\ 1-\frac{1-\omega }{1-\eta_{c}}\frac{\varepsilon_{\max}-\varepsilon_{co}}{\varepsilon_{\max}}-\frac{\varepsilon_{co}}{\varepsilon_{\max}} & & \varepsilon_{c0}<\varepsilon_{\max}\leq \eta_{c}\varepsilon_{c0}\\ 1-\frac{\omega \varepsilon_{c0}}{\varepsilon_{\max}} & & \eta_{c}\varepsilon_{c0}<\varepsilon_{\max}\leq \xi_{c}\varepsilon_{c0}\\ 1 & & \varepsilon_{\max}>\xi_{c}\varepsilon_{c0} \end{array}$$

where ω is the residual compressive strength coefficient; $\varepsilon_{0}$ is the peak strain; η is the residual strain coefficient; λ is the elastic strain coefficient; δ is the elastic compressive strength coefficient; ξ is the limiting strain coefficient; μ is the residual tensile strength coefficient. Subscripts t represent the tension of the element.

The volume fraction of each phase medium can be simplified into area fraction for the two-dimensional random aggregate model. Assuming that the size of the large mesh is *a* and the size of the small mesh is b(a>b), and there are n small mesh element attributes in the large mesh determined as aggregate, then the area fraction of aggregate is $c_{1}=nb_{2}/a_{2}$. Similarly, $c_{0}$, $c_{1}$, $c_{2}$, $c_{3}$ and $c_{4}$ are used to represent the area fraction of new mortar, aggregate, old interface, old mortar and new surface, respectively.

Based on the strain compatibility equation, we obtain the following:

(13) $$\begin{array}{l} \sigma =E_{m}\varepsilon =c_{0}E_{0}\varepsilon_{0}+c_{1}E_{1}\varepsilon_{1}+c_{2}E_{2}\varepsilon_{2}+c_{3}E_{3}\varepsilon_{3}+c_{4}E_{4}\varepsilon_{4}\\ E_{m}=c_{0}E_{0}+c_{1}E_{1}+c_{2}E_{2}+c_{3}E_{3}+c_{4}E_{4} \end{array}$$

The elastic modulus of the parallel equivalent element is as follows:

(14) $$E_{eq}=c_{0}E_{0}{}^{m}(1-D^{m})+c_{1}E_{0}{}^{ag}(1-D^{ag})+c_{2}E_{0}{}^{oitz}(1-D^{oitz})+c_{3}E_{0}{}^{om}(1-D^{om})+c_{4}E_{0}{}^{itz}(1-D^{itz})$$

The thickness of the new and old interface is small for recycled concrete. The mesh element is larger when the homogenized equivalent model is used. A single element may contain multiphase media when the element size is larger than the thickness of the old mortar. Therefore, this article will use recycled concrete as an equivalent to the following three phases: mortar (m), aggregate (ag) and equivalent element (em). The meso-equivalent model of stress–strain relationship for three-phase medium is established, where $\varepsilon_{0}^{\mathrm{em}}\leq \varepsilon_{0}^{m}\leq \varepsilon_{0}^{ag}\leq \varepsilon_{r}^{em}\leq \varepsilon_{r}^{m}\leq \varepsilon_{r}^{ag}\leq \varepsilon_{u}^{em}\leq \varepsilon_{u}^{m}\leq \varepsilon_{u}^{ag}$. The formula of elastic damage model of parallel equivalent element of recycled concrete is as follows:

(15) $$E^{\mathrm{eq}}=\left\{ \begin{array}{l} c_{0}E_{0}^{m}+c_{1}E_{0}^{ag}+c_{2}E_{0}^{em}\;\varepsilon \leq \varepsilon_{0}^{\mathrm{em}}\\ c_{0}E_{0}^{m}+c_{1}E_{0}^{ag}+c_{2}E_{r}^{em}\;\varepsilon_{0}^{em}\text{<}\varepsilon \leq \varepsilon_{0}^{m}\\ c_{0}E_{r}^{m}+c_{1}E_{0}^{ag}+c_{2}E_{r}^{em}\;\varepsilon_{0}^{m}\text{<}\varepsilon \leq \varepsilon_{0}^{ag}\\ c_{0}E_{r}^{m}+c_{1}E_{r}^{ag}+c_{2}E_{r}^{em}\;\varepsilon_{0}^{ag}\text{<}\varepsilon \leq \varepsilon_{r}^{em}\\ c_{0}E_{r}^{m}+c_{1}E_{r}^{ag}+c_{2}E_{u}^{em}\;\varepsilon_{r}^{em}\text{<}\varepsilon \leq \varepsilon_{r}^{m}\\ c_{0}E_{u}^{m}+c_{1}E_{r}^{ag}+c_{2}E_{u}^{em}\;\varepsilon_{r}^{m}\text{<}\varepsilon \leq \varepsilon_{r}^{ag}\\ c_{0}E_{u}^{m}+c_{1}E_{u}^{ag}+c_{2}E_{u}^{em}\;\varepsilon_{r}^{ag}\text{<}\varepsilon \leq \varepsilon_{u}^{em}\\ c_{0}E_{r}^{m}+c_{1}E_{r}^{ag}\;\;\;\varepsilon_{u}^{em}\text{<}\varepsilon \leq \varepsilon_{u}^{m}\\ c_{1}E_{r}^{ag}\;\;\;\;\varepsilon_{u}^{m}\text{<}\varepsilon \leq \varepsilon_{u}^{ag}\\ 0\;\;\;\;\;\;\varepsilon_{u}^{ag}\text{<}\varepsilon \end{array}\right.$$

Formula (15) is the calculation formula of equivalent elastic modulus at each stage. This formula can be obtained by combining the stages in the constitutive model.

Below, we will deduce the calculation formula of equivalent tensile strength of recycled concrete according to energy equivalence. See Formula (16)–(18) for details. From this formula, the equivalent tensile strength of recycled concrete can be calculated. The equivalent element is a homogeneous element. The stored total strain energy W is $W=W_{ag}+W_{em}+W_{om}$ (the sum of the equivalent element, new mortar, and aggregate), when the equivalent element reaches the equivalent tensile strength $f_{t}{}^{eq}$.

Because the strain energy is $W=\int \frac{1}{2}f_{t}^{eq}\varepsilon dV$, where ε is the element strain, the following is obtained:

(16) $$W=W_{ag}+W_{om}+W_{em}=\int \frac{1}{2}f_{t}{}^{ag}\varepsilon dV_{ag}+\int \frac{1}{2}f_{t}{}^{om}\varepsilon dV_{om}+\int \frac{1}{2}f_{t}{}^{em}\varepsilon dV_{em}$$

Substituting $\varepsilon =\frac{f_{t}}{E}$ into the above formula, we obtain the following:

(17) $${\left(f_{t}^{eq}\right)}^{2}E^{eq}V={\left(f_{t}^{ag}\right)}^{2}E^{ag}V_{ag}+{\left(f_{t}^{om}\right)}^{2}E^{om}V_{om}+{\left(f_{t}^{em}\right)}^{2}E^{em}V_{em}$$

(18) $${\left(f_{t}^{eq}\right)}^{2}=E^{eq}\cdot ({\left(f_{t}^{om}\right)}^{2}E^{om}c_{0}+{\left(f_{t}^{ag}\right)}^{2}E^{ag}c_{1}+{\left(f_{t}^{em}\right)}^{2}E^{em}c_{2})$$

**Figure 6:**
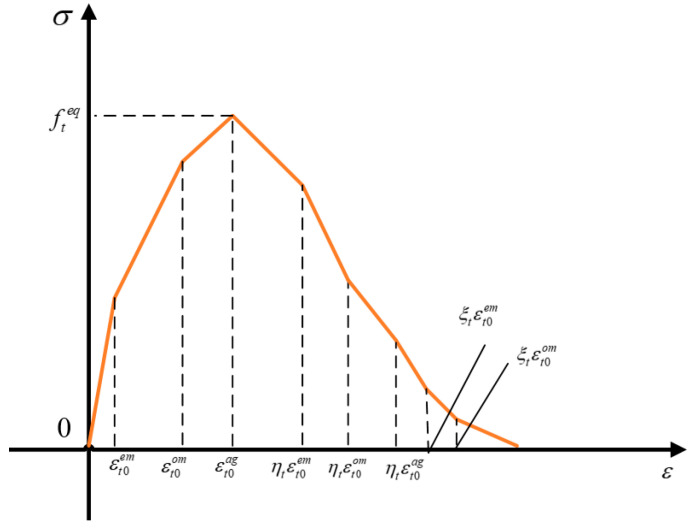
Mean stress/mean strain and damage curves under homogeneous equivalent tensile treatment.

The mean stress/mean strain and damage curves of equivalent element can be obtained after equivalence of different media, as shown in Figure 6.

In Figure 6, the stress–strain curve of homogenized equivalent material is a multi-broken line form. The y-coordinate is the equivalent strength and the x-coordinate is the equivalent strain. After the peak point, there is a period of strength decline, material is damaged and destroyed..

The slope of the curve can be calculated according to Formula (14) and (15).

Zhu et al. [31] assumes that the properties of each component material conform to the Weibull distribution, and considers the non-linear characteristics of material inhomogeneity, thus proposing a random mechanical model. The density function of the Weibull distribution is as follows:

(19) $$f\left(u\right)=\frac{m}{u_{0}}{\left(\frac{u}{u_{0}}\right)}^{m-1}\exp\left[-{\left(\frac{u}{u_{0}}\right)}^{m}\right]$$

where m determines the shape of the Weibull distribution density function, and it represents the uniformity of the medium; u represents a random variable satisfying the Weibull distribution. This paper considers the random distribution of materials in each phase of recycled concrete, and its material parameters obey the Weibull distribution. The value of each parameter is shown in Table 1.

## 3. Calculation Results

### 3.1. Uniaxial Tensile Loading Model

**Figure 7:**
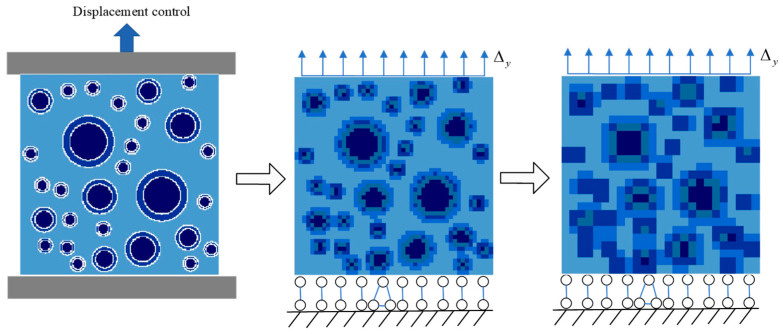
Load model.

The loading model of a cubic recycled concrete specimen is established by using the calculation program of the base force element method of the complementary energy principle in MATLAB programming. The specimen is selected as 100 mm × 100 mm × 100 mm to carry out the uniaxial tensile numerical simulation test. First, the cube model is simplified into a two-dimensional model with a cross-section size of 100 mm × 100 mm, and the loading model is shown in Figure 7. Vertical loading is adopted during loading. A static displacement-controlled loading condition is adopted step-by-step, with a displacement of 0.01 mm for each stage.

### 3.2. Numerical Simulation Results

**Figure 8:**
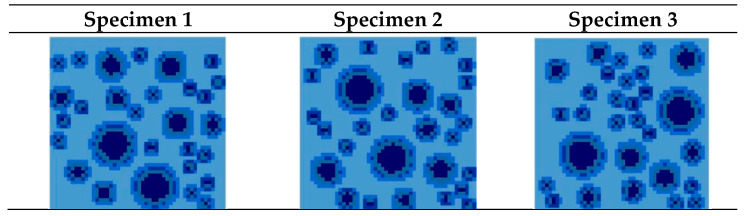
Two-dimensional diagram of homogenized model.

Different random numbers are selected to be put in the aggregate, in order to obtain specimens with different aggregate distribution positions in the random aggregate model. Three two-dimensional numerical models, with three groups of different aggregate distributions and the same aggregate particle number, are selected, as shown in Figure 8.

**Figure 9:**
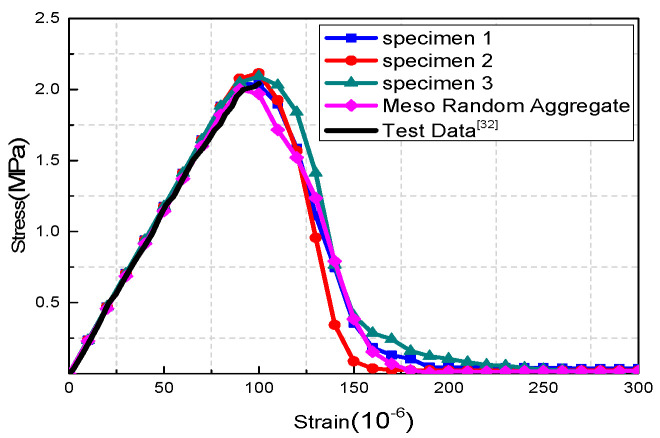
Uniaxial tensile stress–strain curve of parallel model.

The basic force element method based on the principle of complementary energy is used to calculate and analyze the medium damage by the calculation program. The parallel equivalent homogenization model of the generated three random specimens is numerically simulated in a uniaxial tensile test. The calculation results of the three specimens, random aggregate results, and test data [32] are listed in Table 2. Meanwhile, the full stress–strain curve is drawn with strain as the abscissa and stress as the ordinate. The calculation result of the parallel equivalent is shown in Figure 9.

The recycled concrete specimen is in the elastic stage at the initial stage, as shown in Figure 9. The stress begins to grow slowly, and reaches about 80% of the specimen’s ultimate strength. When the strain of the local element is greater than the residual strain, it begins to enter the state of damage. As the strain increases, the stress decreases until it reaches zero.

**Figure 10:**
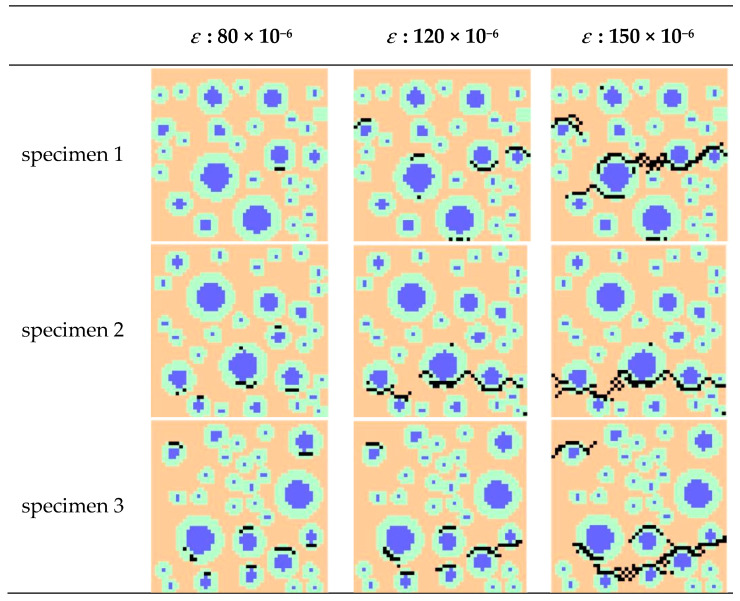
Damage diagram of uniaxial tension numerical simulation.

The QuickWin module in Fortran is adopted to display the different stages of each element with different colors, in order to obtain the damage diagram of the numerical simulation calculation of recycled concrete clearly and simply. The equivalent element will be set as the same yellow–green color, the mortar as orange, the aggregate as blue, and the failure element is represented by the black block. The failure mode of the numerical simulation specimen is observed, as shown in Figure 10.

The law of tensile failure of recycled concrete can be observed from the damage state diagram of specimens in the process of uniaxial tensile loading. The local failure occurs first, when the element begins to be loaded under a certain strain. With the increase in loading strain, cracks gradually spread through the whole specimen, accompanied by the final failure of the specimen. It can be found that the location at which the cracks develop is generally the location where the aggregate is more concentrated in the failure diagram. The main reason for this is that the surrounding strength of the reclaimed aggregate is lower, and it is easier to reach the destruction stage first. The equivalent element contains a multiphase medium, and contains the old interface and the new interface with low tensile strength. Therefore, the element with the cracks that appear first is the equivalent element part. The crack development direction of the tensile failure of the homogenized specimen is perpendicular to the loading direction, and the failure state is basically a horizontal crack, which is consistent with the failure state of the random aggregate model, and is combined with the actual law.

## 4. Discussion

Using the homogenization model of recycled concrete proposed in this paper, the equivalent element is used to replace the tiny random aggregate model element, and the number of available elements is greatly reduced.

In this way, the computing speed is increased and the computer memory is reduced. Table 3 shows the comparison data.

The homogenization model enlarges the size of the grid element of the calculation model, and the equivalent parameters of the homogenization element are obtained by using the parallel equivalent formula.

For the two-dimensional random aggregate recycled concrete model with a size of 100 mm × 100 mm, when the element size of the random aggregate model is 0.5 mm and the element mesh size of the homogenized model is 2 mm, the element mesh size increases by 4 times, the number of elements becomes 1/16 of the original, and the calculation time is reduced by about 300 times.

Obviously, the homogenized equivalent model can greatly save calculation time and improve calculation efficiency, which provides a new way for future numerical simulation analysis and calculation.

## 5. Conclusions

(1) The parallel equivalent stress–strain relationship of the homogenization model is derived. The multi-line damage model of recycled concrete materials is established by using the homogenization analysis method.

(2) The non-linear basic force element analysis software and the homogenization preprocessing software for the homogenization analysis of recycled concrete have been developed, based on the basic force element method of the complementary energy principle.

(3) A parallel-equivalent homogenization model was used to perform a numerical calculation and analysis on the uniaxial tensile test of recycled concrete. The stress–strain softening curve, and the damage and failure process were obtained.

(4) The feasibility and rationality of the model establishment are verified by comparing the results of this method with the experimental data.

(5) The calculation efficiency of the homogenization model has greatly improved. The calculation efficiency of this method is much higher than that of the mesoscopic damage analysis method based on the random aggregate model, and it can guarantee a certain calculation accuracy.

(6) The research work in this paper shows that the base force element method based on the complementary energy principle and the parallel homogenization model can be used to analyze the meso-structure and mechanical properties of recycled concrete. It has the characteristics of high computational efficiency and can be used as an effective meso-analysis method for recycled concrete.

(7) In the future, we will study the base force element method based on the complementary energy principle for dynamic damage analysis and three-dimensional analysis of recycled concrete.

## Figures and Tables

**Table 1:** Material parameter value.

Parameter	New Mortar	Recycled Aggregate	Old Bond Zone	Old Mortar	New Bond Zone
δ	0.25	0.65	0.23	0.25	0.23
ω	0.15	0.15	0.15	0.15	0.15
λ	0.13	0.25	0.15	0.13	0.15
μ	0.3	0.3	0.3	0.6	0.35
$\eta_{c}/\eta_{t}$	4	5	3	4	3
$\xi_{c}/\xi_{t}$	10	10	10	10	10

**Table 2:** Numerical simulation results data.

	Peak Strain (10^−6^)	Peak Stress (MPa)
Test data [32]	102	2.06
Parallel specimen 1	100	2.10
Parallel specimen 2	100	2.11
Parallel specimen 3	100	2.09

**Table 3:** Model data comparison.

Model	Element Size (mm)	Element Number	Calculating Time of One Step (s)
Element of random aggregate model	0.5	40,000	3062
Element of homogenized equivalent model	2	2500	10.8

## Data Availability

The authors declare no conflict of interest.
